# Understanding adverse drug reactions in package leaflets – an exploratory survey among health care professionals

**DOI:** 10.1186/s12913-015-1160-1

**Published:** 2015-11-10

**Authors:** Viktoria Mühlbauer, Ingrid Mühlhauser

**Affiliations:** University of Hamburg, MIN Faculty, Health Sciences and Education, Martin-Luther-King Platz 6, D-20146 Hamburg, Germany

**Keywords:** Drug-related side effects and adverse reactions, Placebo, Drug labelling, Risk communication, Consumer health education

## Abstract

**Background:**

Current German or UK package leaflets do not contain an explicit notice that the listing of side effects does not imply that they are caused by the drug. Causal interpretations by patients and lay people are frequently observed. The authors examined whether health professionals understand that there is not necessarily a causal relation between drug intake and the frequency of side effects and whether adding placebo comparison improves understanding.

**Methods:**

Exploratory survey consisting of eight assessments, each containing 2–6 survey items, and focus groups with one survey sample using questionnaires on adverse reactions in standard package leaflets and modified package leaflets supplemented with placebo comparison. Participants were convenience samples of 379 health professionals including 153 physicians (80 gynaecologists, 124 diabetes experts - physicians, nurses, and others, 39 medical students in their last year at university, 49 first year health science and education students with completed vocational training and professional experience in various health care professions and 87 pharmacists/pharmacy students). They were asked to rate how often the different adverse reactions listed were caused by drug intake.

All surveys were carried out within university seminars and postgraduate lectures from April 2014 to June 2015 in Germany. Response rate was 86 % or higher.

**Results:**

Without placebo comparison, the majority of participants responded that the drug causes adverse reactions with the frequency given in the package leaflet or even more often (95 % of health science students, 100 % of medical students, 60 to 80 % of physicians and 66 % of pharmacists/pharmacy students). Simply adding placebo comparison in a table did not prevent misunderstanding. Analysis of focus groups with health science students supported the lack of understanding.

**Conclusions:**

In the present surveys, health professionals had major difficulties understanding frequency information on side effects in package leaflets. The great majority erroneously implied a causal relation between drug intake and the frequency of side effects, even though most side effects listed are symptoms commonly experienced in daily life.

**Electronic supplementary material:**

The online version of this article (doi:10.1186/s12913-015-1160-1) contains supplementary material, which is available to authorized users.

## Background

At a recent national meeting of an influential women’s health group in Germany, the key participant of a prominent panel discussion on “The Pill” explained why she had stopped taking her oral contraceptives. Studying the package leaflet she was worried by the reported effects of the pill on her psyche. She reasoned: “*More than 1 in 10 experience depression due to the pill*” and “*10 % of women are suffering from tiredness and weakness*” [1]. She undoubtedly interpreted the listed frequencies of symptoms as causally related to the oral contraceptive. We have been observing similar misconceptions among students in health sciences and education. A systematic review of the literature revealed that so far, no research has been published on causal interpretation of adverse events in package leaflets by patients, laypeople or health professionals.

German or UK package leaflets do not contain an explicit notice that side effects listed are not necessarily caused by the drug. However, for estimating a drug’s risk profile it is essential to understand that most side effects can also occur when the specific compound is not taken [2].

There is good evidence that risks and benefits of drugs are over- or underestimated if data on placebo is missing [3, 4]. Therefore, Schwartz et al. developed “drug facts boxes” to communicate drug effects in comparison to placebo. They demonstrated that this format is easily understood by patients, even those with lower education [5]. In the US, some package leaflets already contain placebo information, if the incidence of the adverse reaction is 2 % or more and higher than in the placebo group. Due to the lack of placebo data in German or UK package leaflets drawing conclusions on causality is impossible.

There are no studies explicitly analyzing if health care professionals assume a causal relation for every adverse reaction listed in the package leaflet. One would expect that at least health professionals can correctly interpret drug information. Therefore, the aim of our study was to investigate health professionals’ understanding of the adverse events section of the package leaflet as currently used in countries like Germany or the UK. Secondly, we wanted to evaluate whether understanding can be improved by the additional presentation of placebo data.

## Methods

Exploratory survey consisting of eight assessments with 6 convenience samples of 22 to 124 participants from various health professions including physicians, nurses and other health care professionals, medical students, pharmacists, pharmacy students and students in health sciences and education. A master’s degree in health sciences and education is required to teach at schools for vocational health care professions. Since vocational health care training is required to be admitted to this course, these students have gained professional experience in various health care professions e.g. nursing or doctor’s assistant.

Assessments consisted of a standard or modified package leaflet (mainly excerpts) and a questionnaire. Modified package leaflets contained placebo comparison data. Package leaflets were chosen according to the expected interest of the groups. For further details see Additional file 1, I–VIII.

### Group 1

Twenty-two Year One/second semester students in health sciences and education at Hamburg University. 20/22 (91 %) were female, therefore a package leaflet of combined oral contraceptives was chosen. This group received standard package leaflets only. Answers were given as free text. Two assessments with variations in framing the questions were carried out in April 2014 at 1 week intervals during a seminar on methods of clinical and epidemiological research by one of the authors (IM). Changes in group size are due to students being absent from the seminar.

### Group 2

Twenty-seven Year One/first semester students in health sciences and education at Hamburg University; 25/27 (93 %) were female. As a first assessment they received standard package leaflets, and during a second assessment 1 week later they were presented modified package leaflets of combined oral contraceptives. For this group the questionnaire was supplemented with a question on venous thromboembolism. The presentation of this adverse reaction in package leaflets already includes comparative risk data and was therefore used as a control question. Answers were given as free text. Both assessments were carried out in October 2014 during a seminar in pharmacology held by one of the authors (VM). Changes in group size are due to students being absent from the seminar.

Due to unexpected responses to the modified package leaflet indicating insufficient understanding of comparative data, in depth focus group interviews were conducted with this group a few weeks later on the modified package leaflet containing placebo comparison. The interviews were carried out in four groups of six to seven students using the Think Aloud Test [6]. All interviews were recorded on a voice recorder and transcribed. Analysis was carried out according to Mayring [7].

### Group 3

Eighty gynaecologists at a conference organized by the “German-Turkish Association of Gynaecologists” in the city of Stuttgart in October 2014. This group received only standard package leaflets of combined oral contraceptives. Questions were presented as multiple choice format and answers were given via an electronic response system.

### Group 4

One hundred twenty-four health care professionals during a post-graduate training course in clinical diabetology. Most of them were physicians (59 %) or other health care professionals (e.g. diabetes nurses, 36 %) (5 % others). The training course took place at Friedrich-Schiller-University, Jena, in November 2014. Questions were presented as multiple choice format and answers were given via an electronic response system.

### Group 5

Thirty-nine medical students in their last year at university (fifth year), Martin Luther University, Halle-Wittenberg, Germany. The same standard and modified package leaflet of statins was used for this group as for group 4. Questions were presented as multiple choice format and answers were given in writing. All assessments were carried out in November-December 2014.

### Group 6

Pharmacy group (14 pharmacists, 61 pharmacy students and trainee pharmacists, two other health professionals and ten others) during “Pharmacy day” in June 2015 at the Institute of Pharmacy, Hamburg University. They were presented a standard and a modified package leaflet on statins (Additional file 1, VII). In comparison to group 4 and 5, this time the placebo comparison data were supplemented with *p*-values. *P*-values clearly indicated that the differences in the frequency of side effects between statins and placebo were not statistically significant. Questions were presented as multiple choice format and answers were given via an electronic response system.

Groups 3–6 were asked whether they know or use the Cochrane Library as an indicator of their competencies in evidence-based medicine and risk knowledge. Group 1 had recently been introduced to the Cochrane Library, Group 2 not yet.

In all groups, care was taken to allow for sufficient time and a trustful ambience during the assessments. Participants were invited to support our research project to help improve understandability of package leaflets. Questions were read out loudly with particular emphasis on the word “cause”.

We focused on adverse reactions that seem to occur with a comparable frequency without drug intake – depression/mood disorder and weight gain for oral contraceptives and myalgia/muscle pain for statins. The underlying evidence was taken from two Cochrane reviews [8, 9] and a systematic review [10]. Where Cochrane data were not available we performed a supplementary search for placebo-controlled trials.

In assessments using standard package leaflets, participants were asked to rate how often the drug causes specific symptoms and to estimate how often these symptoms occur without taking the drug (for exact wording see Additional file 1, I–III and V–VII and Tables 1 and 2).

**Table 1:** Responses to questionnaire on standard format package leaflet (oral contraceptives without placebo comparison)

Extract of questionnaire					
Common side effects (more than 1 in 100 people who take Lovelle® are affected):					
• Depression or mood disorder					
• Headache					
• Stomach problems, such as nausea					
• Breast problems, such as painful or tender breasts					
• Weight gain					
Question 1: How often does the intake of this oral contraceptive cause (1) depression or mood disorder (2) weight gain?^a^					
Responses (No)	None	Lower frequency than listed in package leaflet	Same frequency as listed in package leaflet	Higher frequency than listed in package leaflet	No response
Format free text					
Group 1 (*n* = 22) Health science students	(1): 0	(1): 0	(1): 22 (100 %)	(1): 0	(1): 0
	(2): 0	(2): 0	(2): 20 (91 %)	(2): 0	(2): 2 (9 %)
Question 2: If 100 women take this oral contraceptive – in how many women does the intake of this oral contraceptive cause (1) depression or mood disorder (2) weight gain?^b^					
Responses (No)	None	Lower frequency than listed in package leaflet	Same frequency as listed in package leaflet	Higher frequency than listed in package leaflet	No response
Format free text					
Group 1 (*n* = 21) Health science students	(1): 0	(1): 0	(1): 21 (100 %)	(1): 0	(1): 0
	(2): 0	(2): 0	(2): 21 (100 %)	(2): 0	(2): 0
Group 2 (*n* = 25) Health science students	(1): 0	(1): 4 (16 %)	(1): 10 (40 %)	(1): 10 (40 %)	(1): 1 (4 %)
	(2): 0	(2): 3 (12 %)	(2): 10 (40 %)	(2): 11 (44 %)	(2): 1 (4 %)
Question 3: If 100 women take this oral contraceptive – in how many women does the intake of this oral contraceptive cause (1) depression or mood disorder?^b^					
Responses (No)	0	1	>1	>10	No response
Format multiple choice					
Group 3 (*n* = 80) Gynaecologists	(1): 0	(1): 11 (14 %)	(1): 56 (70 %)	(1): 7 (9 %)	(1): 6 (7 %)
Question 4: If 100 women do not take an oral contraceptive – in how many of them does (1) depression or mood disorder (2) weight gain occur?^c^					
Responses (No)	None	Lower frequency than listed in package leaflet	Same frequency as listed in package leaflet	Higher frequency than listed in package leaflet	No response
Format free text					
Group 1 (*n* = 21) Health science students	(1): 0	(1): 12 (57 %)	(1): 1 (5 %)	(1): 1 (5 %)	(1): 7 (33 %)
	(2): 0	(2): 12 (57 %)	(2): 1 (5 %)	(2): 1 (5 %)	(2): 7 (33 %)

^a^Possible answers: Since the exact value cannot be known from the information provided, any value lower than given in the package leaflet would be accepted. The frequency given in the package leaflet or higher would definitely be wrong. Due to it being free text, missing responses were seen as a sign of non-participation

^b^Since the exact value cannot be known from the information provided, any value lower than given in the package leaflet including no response would be accepted. The frequency given in the package leaflet or higher would definitely be wrong

^c^Possible answers: Since the exact value cannot be known from the information provided, any value except “none” or higher values than listed in the package leaflet would be accepted. Due to it being free text, missing responses were seen as a sign of non-participation

**Table 2:** Responses to questionnaire on standard format package leaflet (statins without placebo comparison)

Extract of questionnaire					
CRESTOR® 10 mg film-coated tablets					
Common possible side effects (these may affect between 1 in 10 and 1 in 100 patients):					
• Headache					
• Stomach pain					
• Constipation					
• Feeling sick					
• Muscle pain					
• Feeling weak					
• Dizziness					
Question 1: If 100 patients take this statin – in how many of them does the intake of this statin cause muscle pain?^a^					
Responses (No)	0	1	>1	>10	No response
Format multiple choice					
Group 4 (*n* = 124) Diabetes experts			75^b^ (60 %)		
Group 5 (*n* = 39) Medical students	0	0	37 (95 %)	2 (5 %)	0
Group 6 (*n* = 87) Pharmacy group	2 (2 %)	9 (10 %)	57 (66 %)	19 (22 %)	0
Question 2: If 100 patients do not take this statin – in how many of them does muscle pain occur?^c^					
Responses (No)	0	1	>1	>10	No response
Format multiple choice					
Group 4 (*n* = 124) Diabetes experts	16 (13 %)	11 (9 %)	64 (51 %)	17 (14 %)	16 (13 %)
Group 5 (*n* = 39) Medical students	24 (62 %)	5 (13 %)	4 (10 %)	5 (13 %)	1 (2 %)
Group 6 (*n* = 87) Pharmacy group	18 (21 %)	8 (9 %)	35 (40 %)	25 (29 %)	1 (1 %)

^a^Since the exact value cannot be known from the information provided, any value lower than given in the package leaflet including no response would be accepted. The frequency given in the package leaflet or higher would definitely be wrong

^b^Exact data lost due to technical problems, but the value was confirmed independently by two other lecturers

^c^Possible answers: Since the exact value cannot be known from the information provided, any value except “none” and “more than 10” including no response would be accepted

In order to find out whether adding the frequency with which specific symptoms occur under placebo treatment [11] would improve understanding we supplemented standard package leaflet information by a table displaying frequencies of adverse reactions under drug treatment in comparison to placebo treatment. All frequencies were given as percentages only – a format usually well understood [12].

Participants were considered to be misunderstanding the package leaflet when reported values were equal to the frequency in the package leaflet or higher. Answers were accepted if they were “none” or at least of a lower frequency than stated in the package leaflet. In multiple choice questions, missing answers could be an answer as well as a sign of non-participation.

According to the literature, a causal relationship between the chosen drugs and the selected adverse reactions does not exist [8, 9, 11]. Still, the underlying evidence base is insufficient. A causal relation can therefore neither be confirmed nor completely ruled out.

### Ethics statement

Our survey complied with the Helsinki Declaration. According to the code of medical ethics for Hamburg, obtaining formal ethical approval for this study was not required. No medical interventions were involved and the impact of the questionnaires on daily life was considered minor and thus the welfare and rights of the participants were protected. Participants were free to answer the questions or not. Students were told that the survey was anonymous and had no impact on the grading of the seminar. The survey was conducted according to German data privacy regulations.

## Results

### Standard package leaflet

When asked how often the oral contraceptive causes depression or weight gain, 22/22 (100 %) (depression) and 20/22 (91 %) (weight gain) health science students (group 1) stated the frequency of occurrence given in the package leaflet (Table 1/question 1). Slightly rephrasing the question resulted in identical answers 1 week later (Table 1/question 2). Of the second group of health science students (group 2) who had just entered university, 10/25 (40 %) (mood disorder and weight gain) answered within the frequency given in the package leaflet, whereas 10 to 11/25 (40–44 %) even replied these side effects were caused more often by the oral contraceptive than the frequency listed in the package leaflet (Table 1/question 2). None of them replied that questions on causality could not be answered based on the information provided. However, 18, 21 and 22 of the 25 students were able to correctly report the frequencies of venous thrombosis as reported in three scenarios in the package leaflet (see Additional file 1, III).

Similarly, 56/80 (70 %) gynaecologists (group 3) rated the frequency given in the package leaflet and 7/80 (9 %) even higher (Table 1/question 3). Only 11/80 (14 %) participants responded with a lower frequency (6/80 (7 %) no response).

When asked how many women *not* taking any oral contraceptives experience the symptoms depression and weight gain, 12/21 (57 %) (group 1, Table 1/question 4) believed that these symptoms must occur less often than in women taking oral contraceptives,”otherwise it would not be listed as side effect in the package leaflet“.

Comparable results were obtained using the original statin package leaflet; 75/124 (60 %) participants of the post-graduate training in clinical diabetology (group 4) replied that the statin causes myalgia or muscle pain with the given frequency of occurrence listed in the package leaflet (Table 2/question 1, exact data lost due to technical problems, but the value was confirmed independently by two other lecturers).

Results were similar for fifth year medical students (group 5) and the pharmacy group (group 6). None of the medical students and only 11/87 (12 %) of the pharmacy group responded a value lower than listed in the package leaflet (Table 2/question 1). 24/39 (62 %) of the medical students and 18/87 (21 %) of the pharmacy group stated that muscle pain would not occur in any patient not taking statins (Table 2/question 2).

### Modified package leaflet

Adding data on the occurrence of side effects in the placebo group to the package leaflet on oral contraceptives didn’t have a substantial effect. None of the health science students (group 2) realised that there was no difference between the groups (Table 3/question 1).

**Table 3:** Responses to questionnaire on modified package leaflet (oral contraceptives with placebo comparison)

Extract of questionnaire					
Frequency of side effects that were reported in a clinical trial on norgestimate/ ethinylestradiol (e.g. Amicette®) with 462 participants:					
		Norgestimate/ethinylestradiol		Placebo	
Headache		18.4 %		20.5 %	
Painful or unusual periods		10.1 %		9.0 %	
Weight gain		2.2 %		2.1 %	
Question 1: If 100 women take this oral contraceptive – in how many women does the intake of this oral contraceptive cause (1) headache (2) painful or unusual periods (3) weight gain?^a^					
Responses (No)	None	Lower frequency than listed in package leaflet	Same frequency as listed in package leaflet	Higher frequency than listed in package leaflet	No response
Format free text					
Group 2 (*n* = 27) Health science students	0	(1): 7 (26 %)	(1): 12 (44 %)	(1): 3 (11 %)	(1): 5 (19 %)
	0	(2): 6 (22 %)	(2): 13 (48 %)	(2): 6 (22 %)	(2): 2 (7 %)
	0	(3): 7 (26 %)	(3): 13 (48 %)	(3): 2 (7 %)	(3): 5 (19 %)
Question 2: If 100 women do not take an oral contraceptive – in how many of them does (1) headache (2) painful or unusual periods (3) weight gain occur?^b^					
Responses (No)	None	Lower frequency than listed in package leaflet	Same frequency as listed in package leaflet	Higher frequency than listed in package leaflet	No response
Format free text					
Group 2 (*n* = 27) Health science students	0	(1): 7 (26 %)	(1): 12 (44 %)	(1): 3 (11 %)	(1): 5 (19 %)
	0	(2): 6 (22 %)	(2): 13 (48 %)	(2): 4 (15 %)	(2): 4 (15 %)
	0	(3): 6 (22 %)	(3): 13 (48 %)	(3): 3 (11 %)	(3): 5 (19 %)

^a^Possible answers: “None” is the correct answer. Since we have not provided any *p*-values for this table and therefore statistical significance cannot be identified, “1” would also be correct in part (2) and accepted in (3). Due to it being free text, missing responses were seen as a sign of non-participation

^b^Possible answers: Whether women not taking oral contraceptives are equal to women taking placebo depends on placebo and nocebo effects. Therefore, all answers except “none” were accepted. Due to it being free text, missing responses were seen as a sign of non-participation

Analysis of the focus group interviews revealed that the students had difficulties understanding the slightly more scientific data. Most students were unable to make use of the frequency of occurrence presented only as percentages. Furthermore, being given the total number of patients participating in the study greatly confused them. However, they were able to understand placebo data to be roughly comparable to people not on medication and to find the corresponding values in the package leaflet (Table 3/question 2).

When the participants of the post-graduate training in clinical diabetology (group 4) were presented with the statin comparison to placebo data, still 65/124 (52 %) answered the frequency as originally given in the package leaflet and 9/124 (7 %) even higher. Only 16/124 (13 %) realised that there was no difference (Table 4).

**Table 4:** Responses to questionnaire on modified package leaflet (statins with placebo comparison)

Extract of questionnaire					
Frequency of side effects that were reported in clinical trials on statins with 37,938 patients (excerpt, Cochrane review 2013):					
	Statins		Placebo		
Muscle pain	9.5 %		9.2 %		
Question: If 100 patients take this statin – in how many of them does the intake of this statin cause muscle pain?^a^					
Responses (No)	0	1	>1	>10	No response
Format multiple choice					
Group 4 (*n* = 124) Diabetes experts	16 (13 %)	17 (14 %)	65 (52 %)	9 (7 %)	17 (14 %)
Group 5 (*n* = 39) Medical students	6 (15 %)	6 (15 %)	22 (57 %)	4 (10 %)	1 (3 %)

^a^Possible answers: “0” would be the correct answer. Since we have not provided any *p*-values for this table and therefore statistical significance cannot be identified, “1” would also be accepted

Despite the placebo comparison, at least 26/39 (67 %) medical students (group 5) were unable to see that there is no difference in occurrence between the two groups (Table 4). Results in the pharmacy group (group 6) were similar (Table 5), although they had been provided with the corresponding *p*-values indicating non-significance: 77/87 (88 %) did not see that muscle pain occurred as often in the placebo group as in the statin group.

**Table 5:** Responses to questionnaire on modified package leaflet supplemented with *p*-values (statins with placebo comparison)

Extract of questionnaire					
Frequency of muscle pain that was reported in clinical trials on statins:					
	Statins		Placebo		*p*-value
Cochrane 2013 *N* = 37,938, primary prevention	9.5 %		9.2 %		0.40
Finegold 2014 *N* = 46,262, primary prevention	7.9 %		7.6 %		0.407
Finegold 2014 *N* = 37,618, secondary prevention	4.8 %		4.6 %		0.558
Question: If 100 patients take this statin – in how many of them does the intake of this statin cause muscle pain?^a^					
Responses (No)	0	1	>1	>10	No response
Format multiple choice					
Group 6 (*n* = 87) Pharmacy group	10 (12 %)	12 (14 %)	48 (55 %)	14 (16 %)	3 (3 %)

^a^Possible answers: “0” would be the correct answer

## Discussion

Our survey indicates that various health care professionals have major difficulties understanding frequency information on adverse events in package leaflets. The great majority erroneously implied a causal relation between drug intake and the rate with which the listed symptoms occur. Supplementation with placebo data did not prevent misinterpretation.

Package leaflets have always been a target of criticism and subject to change. Three decades ago, they were still optional, used medical terminology and contained only verbal descriptors to classify adverse reactions [13, 14]. Nowadays, package leaflets are mandatory, more user-friendly, and numerical frequencies have become an obligatory supplement to verbal descriptors [15, 16]. Placebo information is usually not included, although information on comparators has been supplemented for selected risks. For example, in an increasing number of package inserts of oral contraceptives, thromboembolic risk is now communicated for women who take this pill, those who do not take it or take a pill with a different component, and pregnant women.

So far, most research on patients’ understanding of the reported adverse events has been done on direct-to-consumer advertising. Davis [17] had analysed potential customers’ preferences as to whether placebo data should be presented in drug advertisements. Of the 364 participants in the survey, 52.6 % preferred the inclusion of placebo-group incidence levels, whereas 37.5 % did not, 9.9 % had no preferences. However, wanting the information does not necessarily mean that the information is understood. O’Donoghue [4] had used placebo comparison for quantifying only benefit information in drug advertisements. Therefore the results may not be transferable to risk information. They found that placebo rate information resulted for some participants in more accurate benefit perceptions whereas benefit was greatly overestimated when placebo information was missing.

Tan et al. pointed out that the “excessive, inconsistent and poorly presented information about adverse drug reactions” [2] has the potential to do harm. Most adverse drug reactions listed in package leaflets and other sources of drug information are unspecific symptoms commonly experienced in daily life. Patients assuming a causal relation between drug intake and every adverse reaction listed in the package leaflet may substantially overestimate harm caused by the drug and thus decide against intake for the wrong reason [2]. Pfistermeister et al. drew comparable conclusions. Furthermore, they found that up to 40 % of UK and German package inserts contain “at least one potentially conflicting pair of adverse reactions”, i.e. contradictory symptoms like increased and decreased appetite [18].

In our study, just presenting percentages of adverse events during drug treatment compared to placebo treatment in a table format did not enable health care professionals to question the assumed causal relationship between drug intake and the rate of occurrence of listed side effects. Even physicians and medical students seem to have great difficulties understanding placebo comparisons.

Difficulties seem to be related to understanding and interpreting data, not to finding data within the package leaflet or reading tables. Tables that demand no particular risk literacy and additional interpretation could be subject of further research.

Various factors and the use of convenience samples itself can adversely interfere with the performance of surveys leading to biased findings. Therefore, we took great care to offer a trustful ambience and the necessary time frame. Participation rates were high, and all 6 heterogeneous survey groups gave similar responses. Different framing of questions gave identical responses. Notably, the majority of health science students correctly identified comparative risk information for thromboembolic events from the package leaflets of oral contraceptives. Analysis of the focus group interviews supported the overall findings indicating a lack of understanding of placebo data. Therefore, we are confident that the results of our survey are valid.

Our study has limitations. The survey has an explorative design. It was carried out in Germany including convenience samples. Only a small proportion of participants indicated that they used the Cochrane Library, a larger group didn’t even know it (Additional file 1, VIII). Therefore, competencies in evidence-based medicine and risk knowledge might be higher in other countries possibly also facilitating better understanding of package leaflets. In order to achieve high participation rates we refrained from collecting further demographic information or assessing numeracy.

Finally, in order to attract the attention of our survey participants, the questions on oral contraceptives and statins dealt with side effects that are commonly attributed to the drug, but where evidence does not unambiguously support a causal relationship. In fact, there were no significant differences in the drug and placebo comparisons used in our examples. We are aware of the controversies surrounding myalgia and muscle pain during statin treatment [19], and it is difficult to identify studies comparing oral contraceptives with placebo reporting statistically significant differences in side effects. We could not identify evidence on there being a causal relation between the two [8, 11]. However, it might also be due to insufficient reporting of adverse events in clinical trials [20]. Despite this uncertainty, it is improbable that depression and mood disorder during oral contraceptive use are caused with a frequency of 1–10 % as listed in most package leaflets. The focus of our study was to analyse understanding. Therefore, the limitations related to the evidence base used in our examples can be neglected - an approach equally used by Schwartz et al. [5] to evaluate the drug-facts-boxes.

Despite the limitations of this explorative study, we doubt that investigations including samples more representative for various health care professionals would provide different results. Rather, the focus of further research should be the development of a format informing about side effects that can easily be understood.

## Conclusion

Currently, the package leaflet is first and foremost a legal document rather than an instrument to inform patients – the information provided in the package leaflet is regulated by law [21] and the pharmaceutical company can be sued if damage occurred due to a package leaflet not being on the current state of medical knowledge [22]. It is therefore not surprising that pharmaceutical companies add side effects to the package leaflet whenever there is the slightest possibility for a causal relation. Still, our analysis shows that even the majority of physicians and other health care professionals assume a causal relation between drug intake and the side effects listed in the package leaflet and therefore don’t understand the information provided. Thus, it is improbable that they inform patients appropriately on the risk of adverse events due to drug administration. Informed decision-making is then impossible. Although we acknowledge that package leaflets do not need to and cannot be a decision aid, the information provided must be understandable. Other ways of presenting data on adverse events have to be found [23] – demanding quite a balancing act between legal requirements and understandable patient information.

## Additional file

Additional file 1:
**PDF-document (.pdf), questionnaires and supplementary data on convenience samples and their knowledge on EbM.** (PDF 272 kb)
