# Supplementary material for: Understanding adverse drug reactions in package leaflets – an exploratory survey among health care professionals
Source: BMC Health Serv Res. 2015 Nov 10;15:505. doi: 10.1186/s12913-015-1160-1 (PMC4641349; doi:10.1186/s12913-015-1160-1)
Supplement: Additional file 1: — PDF-document (.pdf), questionnaires and supplementary data on convenience samples and their knowledge on EbM. (PDF 272 kb) [file 12913_2015_1160_MOESM1_ESM.pdf]

Supplementary material 1

All questionnaires were originally in German. The excerpts from package inserts found in this supplement are closely translated to the German original. They might therefore differ from the official English/UK package inserts.

I. First assessment with first-year students in health science and education (group 1). Students could bring the package insert of their own oral contraceptive or were provided the complete package insert of Lovelle®.

__Questionnaire__

Dear participants,

in preparation of the seminar on case-control studies where we will read an article on oral contraceptives, we would like to analyze the understandability of package inserts of oral contraceptives. The aim is to identify weaknesses of package inserts in order to define potential improvements. The survey is anonymous.

Please try to find the information in the package insert and try to give your responses in numbers with reference parameters. If the exact information cannot be found, please indicate your best possible estimate:

Package leaflet:
Information for the user
Lovelle®
Desogestrel and Ethinyl estradiol

4. Possible side effects

Like with all medicines, Lovelle® can have side effects, although not everybody gets them.

Tell your doctor or pharmacist if you get any side effects particularly if they are severe and persistent, or you have any change in your health which you think may be due to Lovelle®.

Common side effects (more than 1 in 100 people who take Lovelle® are affected):
• Depression or mood disorder<br>• Headache<br>• Stomach problems, such as nausea<br>• Breast problems, such as painful or tender breasts<br>• Weight gain

Side effects (can usually be found under “4.” in package inserts):

a) How often does the intake of this oral contraceptive cause depression or mood disorder?

b) How often does the intake of this oral contraceptive cause weight gain?

II. Second assessment on standard package inserts for first year students in health science and education (group 1) one week later.

<u>Questionnaire</u>

Dear participants,

we would like to analyze the understandability of package inserts. Today, the focus is on the presentation of side effects. The survey is anonymous.

Please try to find the information in the package insert and try to give your responses in numbers with reference parameters. If the exact information cannot be found, please indicate your best possible estimate:

Package leaflet:
Information for the user
Lovelle®
Desogestrel and Ethinyl estradiol

4. Possible side effects

Like with all medicines, Lovelle® can have side effects, although not everybody gets them.

Tell your doctor or pharmacist if you get any side effects particularly if they are severe and persistent, or you have any change in your health which you think may be due to Lovelle®.

Common side effects (more than 1 in 100 people who take Lovelle® are affected):
• Depression or mood disorder<br>• Headache<br>• Stomach problems, such as nausea<br>• Breast problems, such as painful or tender breasts<br>• Weight gain

1)

a) If 100 women take this oral contraceptive – in how many women does the intake of this oral contraceptive cause depression or mood disorder?
b) If 100 women do not take an oral contraceptive – in how many of them does depression or mood disorder occur?

2)

a) If 100 women take this oral contraceptive – in how many women does the intake of this oral contraceptive cause weight gain?
b) If 100 women do not take an oral contraceptive – in how many of them does weight gain occur?

III. First assessment on standard package inserts for first year students in health science and education (group 2).

<u>Questionnaire</u>

Dear participants,

we would like to analyze the understandability of package inserts. Today, the focus is on the presentation of side effects. The survey is anonymous.

Please try to find the information in the package insert and try to give your responses in numbers with reference parameters. If the exact information cannot be found, please indicate your best possible estimate:

Package leaflet:
Information for the user
Lovelle®
Desogestrel and Ethinyl estradiol

2.2.1 Oral contraceptives and risk of venous thrombosis

	**Risk of venous thrombosis per year**
Women who are **not** on the Pill and not pregnant	Approximately 2 out of 10,000 women
Women who use an oral contraceptive containing **levonorgestrel, norethisteron** or **norgestimate**	Approximately 5-7 out of 10,000 women
Women who take Lovelle®	Approximately 9-12 out of 10,000 women

4. Possible side effects

Like with all medicines, Lovelle® can have side effects, although not everybody gets them.

Tell your doctor or pharmacist if you get any side effects particularly if they are severe and persistent, or you have any change in your health which you think may be due to Lovelle®.

Common side effects (more than 1 in 100 people who take Lovelle® are affected):
• Depression or mood disorder<br>• Headache<br>• Stomach problems, such as nausea<br>• Breast problems, such as painful or tender breasts<br>• Weight gain

1) Side effects

a) If 100 women take this oral contraceptive – in how many women does the intake of this oral contraceptive cause depression or mood disorder?

b) If 100 women take this oral contraceptive – in how many women does the intake of this oral contraceptive cause weight gain?

2) Risk of venous thrombosis:

a) How often do venous thromboses occur in women who are on this oral contraceptive for one year?

b) How often do venous thromboses occur in women who are on another oral contraceptive?

c) How often do venous thromboses occur in women who don't take any oral contraceptive?

Responses to 2)

	number of correct responses	Missing
a)	18/25	0
b)	22/25	0
c)	21/25	0

IV. Second assessment with modified package insert for first-year students health science and education (group 2) one week later.

<u>Questionnaire</u>

Dear participants,

we would like to analyze the understandability of package inserts. Today, the focus is on the presentation of side effects. The survey is anonymous.

Please try to find the information in the package insert and try to give your responses in numbers with reference parameters.

PACKAGE LEAFLET: INFORMATION FOR THE USER

Amicette®

Norgestimate/ethinylestradiol

1. What Amicette® does

Amicette® is a combined hormonal contraceptive pill ('the Pill'). You take it to stop getting pregnant. This contraceptive contains two types of female sex hormones, oestrogen and progestogen. Therefore, Amicette® is called a combined oral contraceptive.

4. Possible side effects

Like all medicines, Amicette® can have side effects, although not everybody gets them.
Tell your doctor, pharmacist or health care professional if you are worried about any side effects which you think may be due to Amicette®. Tell your doctor if you notice any other effects that are not listed below.

Frequency of side effects that were reported in a clinical trial on norgestimate/ ethinylestradiol (e.g. Amicette®) with 462 participants (excerpt):

	Norgestimate/ethinylestradiol	Placebo
Headache	18.4%	20.5%
Painful or unusual periods	10.1%	9.0%
Weight gain	2.2%	2.1%

1)

a) If 100 women take this oral contraceptive – in how many women does the intake of this oral contraceptive cause painful or unusual periods?

b) If 100 women do not take an oral contraceptive – in how many of them do painful or unusual periods occur?

2)

a) If 100 women take this oral contraceptive – in how many women does the intake of this oral contraceptive cause weight gain?

b) If 100 women do not take an oral contraceptive – in how many of them does weight gain occur?

3)

a) If 100 women take this oral contraceptive – in how many women does the intake of this oral contraceptive cause headaches?

b) If 100 women do not take an oral contraceptive – in how many of them do headaches occur?

V. Questionnaire for gynaecologists at the autumn conference in Stuttgart organized by the German-Turkish association of gynaecologists in October 2014.

A)

Lovelle®
Desogestrel and Ethinyl estradiol

4. Possible side effects

Like with all medicines, Lovelle® can have side effects, although not everybody gets them.

Common side effects (more than 1 in 100 people who take Lovelle® are affected):
• Depression or mood disorder<br>• Headache<br>• Stomach problems, such as nausea<br>• Breast problems, such as painful or tender breasts<br>• Weight gain

Question 1: If 100 women take this oral contraceptive – in how many women does the intake of this oral contraceptive cause depression or mood disorder?

A: 0 B: 1 C: >1 D: >10

B)
The Cochrane Library...

A I use the Cochrane Library
B I know the Cochrane Library, but I don't use it
C I don't know the Cochrane Library yet

VI. Questionnaire on standard and modified package insert used for fifth year medical students from Martin-Luther-University, Halle-Wittenberg and diabetes experts at a postgraduate training course in clinical diabetology in Jena, November – December 2014.

A)
CRESTOR® 10mg film-coated tablets
Common possible side effects (these may affect between 1 in 10 and 1 in 100 patients):

- Headache
- Stomach pain
- Constipation
- Feeling sick
- Muscle pain
- Feeling weak
- Dizziness

Question1: If 100 patients take this statin – in how many of them does the intake of this statin cause muscle pain?

A: 0 B: 1 C: >1 D: >10

Question 2: If 100 patients do **not** take this statin – in how many of them does the intake of this statin cause muscle pain?

A: 0 B: 1 C: >1 D: >10

B)

Frequency of muscle pain that were reported in clinical trials with 37938 patients (excerpt, Cochrane Review 2013)::		
	Statins	Placebo
Muscle pain	9.5%	9.2%

Question 3: If 100 patients take this statin – in how many of them does the intake of this statin cause muscle pain?

A: 0 B: 1 C: >1 D: >10

C)
The Cochrane Library...

A I use the Cochrane Library
B I know the Cochrane Library, but I don't use it
C I don't know the Cochrane Library yet

VII. Questionnaire on standard and modified package insert used for pharmacists and pharmacy students, lecture during “pharmacy day” Hamburg University, June 2015.

A)
CRESTOR® 10mg film-coated tablets
Common possible side effects (these may affect between 1 in 10 and 1 in 100 patients):

- Headache
- Stomach pain
- Constipation
- Feeling sick
- Muscle pain
- Feeling weak
- Dizziness

Question1: If 100 patients take this statin – in how many of them does the intake of this statin cause muscle pain?

A: 0 B: 1 C: >1 D: >10

Question 2: If 100 patients do **not** take this statin – in how many of them does the intake of this statin cause muscle pain?

A: 0 B: 1 C: >1 D: >10

B)

Frequency of muscle pain that was reported in clinical trials on statins:			
	Statins	Placebo	p-value
Cochrane 2013<br>N=37938, primary prevention	9.5%	9.2%	0.40
Finegold 2014<br>N=46262, primary prevention	7.9%	7.6%	0.407
Finegold 2014<br>N=37618, secondary prevention	4.8%	4.6%	0.558

Question 3: If 100 patients take this statin – in how many of them does the intake of this statin cause muscle pain?

A: 0 B: 1 C: >1 D: >10

C)
The Cochrane Library...

A I use the Cochrane Library
B I know the Cochrane Library, but I don’t use it
C I don’t know the Cochrane Library yet

VIII. Responses to question on Cochrane Library

The Cochrane Library...

A I use the Cochrane Library
B I know the Cochrane Library, but I don’t use it
C I don’t know the Cochrane Library yet

1) Group 3: Gynaecologists (n=80)

A	B	C	No response
5	52	15	8

2) Group 4: Participants of postgraduate course in clinical diabetology (n=124)

A	B	C	No response
23	54	30	17

3) Group 5: Medical students (fifth year) (n=39)

A	B	C	No response
0	10	29	0

4) Group 6: Pharmacists and pharmacy students (n=87)

A	B	C	No response
2	16	67	2
